# Subchronic PAH Exposure Impacts Cardiac Electrophysiology of the Polar Fish, Navaga Cod (*Eleginus nawaga*)

**DOI:** 10.3390/toxics14080674

**Published:** 2026-07-30

**Authors:** Irina Dzhumaniiazova, Tatiana S. Filatova, Artem V. Shamshura, Denis V. Abramochkin

**Affiliations:** 1 Department of Human and Animal Physiology, Faculty of Biology, Lomonosov Moscow State University, 119234 Moscow, Russia; dzhumaniiazova.irina@gmail.com (I.D.);; 2 Institute of Physiology, Pirogov Russian National Research Medical University, Ostrovityanova Str., 117513 Moscow, Russia

**Keywords:** polycyclic aromatic hydrocarbons, phenanthrene, 3-methylphenanthrene, cardiomyocytes, patch-clamp, ionic currents, action potential, fish heart

## Abstract

Polycyclic aromatic hydrocarbons (PAHs) from the water-soluble fraction (WSF) of crude oil are recognized cardiotoxicants in fish; however, all electrophysiological evidence to date derives from acute exposure experiments. This study examined the effects of prolonged (5–8 h) incubation of isolated ventricular cardiomyocytes from the navaga cod (*Eleginus nawaga*, Walbaum, 1792) with 10% WSF (total dissolved tricyclic-PAH concentration in the tens-of-nM), 3 µM phenanthrene (Phe), or 3 µM 3-methylphenanthrene (3-MP) on action potential (AP) parameters and the underlying ionic currents using the whole-cell patch-clamp technique. WSF and 3-MP reduced AP amplitude and maximum rate of depolarization through suppression of the fast sodium current (I_Na_), whereas Phe and 3-MP shortened AP duration, associated with decreased density and a hyperpolarizing shift in steady-state activation of the rapid delayed rectifier potassium current (I_Kr_). The L-type calcium current and inward rectifier potassium current remained unaffected by all treatments. Notably, the effects of prolonged incubation differed qualitatively from previously reported acute responses to the same compounds, suggesting the involvement of indirect mechanisms such as reactive oxygen species production and activation of intracellular signaling cascades. These findings demonstrate that subchronic PAH exposure produces distinct and potentially proarrhythmogenic alterations in fish cardiac electrophysiology that cannot be predicted from acute exposure data alone.

## 1. Introduction

Crude oil extraction and transport carry substantial and growing environmental risks. Routine spills typically do not attract public attention; however, by some estimates, their cumulative volume may reach 1–7% of annual production. Each year, up to 1 million tonnes of petroleum products enter the water systems of the major Siberian rivers—the Ob, Yenisei, and Lena—and are subsequently transported into the seas of the Arctic basin [1]. According to the European Environment Agency, routine discharges contribute approximately 1–3 megatonnes of oil to the World Ocean annually [2]. Thus, the cumulative contribution of regular leaks arising from minor pipeline accidents, extraction operations, and tanker transport substantially exceeds the volume of oil entering the hydrosphere as a result of rare but highly publicized catastrophic spills. Oil entering aquatic environments inflicts considerable ecological damage, and marine organisms are among the most vulnerable to its chronic effects.

The toxicity of the water-soluble fraction (WSF) of crude oil was investigated most thoroughly following the Exxon Valdez tanker disaster, which led to elevated mortality in Pacific herring and pink salmon inhabiting the affected region, as well as an increased incidence of developmental abnormalities in fish [3]. Subsequent studies demonstrated that developmental impairments in fish larvae exposed to WSF are invariably preceded by cardiac dysfunction, including atrioventricular conduction block and bradycardia [4]. These findings indicate that the effects of WSF are primarily cardiotoxic. Indeed, electrophysiological studies conducted on isolated ventricular cardiomyocytes of Atlantic bluefin tuna (*Thunnus thynnus*) and yellowfin tuna (*Thunnus albacares*) were the first to demonstrate that WSF suppresses the amplitude of the rapid delayed rectifier potassium current (I_Kr_), which is responsible for myocardial repolarization in teleost fish. The reduction in I_Kr_ amplitude resulted in prolongation of the action potential (AP) duration. The amplitude of the calcium current, which is critically important for excitation–contraction coupling in these cells, was also decreased in a dose-dependent manner in the presence of WSF [5]. This pioneering work initiated intensive study of the cardiotoxic effects of individual PAHs across fish species.

Crude oil is a complex mixture of organic compounds; however, the effects of WSF derived from different crude oil grades have proven to be remarkably similar, suggesting that the observed toxic effects are mediated by a limited set of water-soluble constituents common to all grades. Polycyclic aromatic hydrocarbons (PAHs)—a broad class of organic compounds containing two or more fused benzene rings—have been proposed as the principal toxic agents responsible for these effects.

Dicyclic PAHs have relatively weak direct effects: naphthalene (5 µM) did not affect calcium transients in mackerel (*Scomber japonicus*) ventricular myocytes, whereas phenanthrene at the same concentration significantly reduced them and inhibited both I_Ca,L_ and I_Kr_ in bluefin tuna, prolonging the AP [6]. Over the past decade, attention has therefore focused on the species-specific effects of tricyclic PAHs.

Phenanthrene and its derivative, retene, were studied first, as the most abundant tricyclic constituents of WSF. In rainbow trout (*Oncorhynchus mykiss*), retene significantly increased AP amplitude and shortened AP duration already at 1 µM, whereas phenanthrene shortened the AP only at 10–30 µM; 1 µM retene and 10 µM phenanthrene increased peak I_Na_ but reduced both I_Ca_ and I_Kr_ [7]. These effects are species-dependent: in bluefin tuna, phenanthrene prolonged the AP, whereas in rainbow trout, it shortened it.

Phenanthrene prolonged the AP in ventricular myocytes of brown trout (*Salmo trutta*) and shorthorn sculpin (*Myoxocephalus scorpio*), reflecting the high sensitivity of I_Kr_ (IC50 7.2 µM and 1.4 µM, respectively). Depolarizing currents were also affected, with 10 µM phenanthrene suppressing >50% of peak I_Na_ in sculpin and 30 µM reducing I_Ca_ in trout cardiomyocytes by ~30% [8,9]. In zebrafish (*Danio rerio*), in contrast, 3 µM phenanthrene shortened the AP through preferential inhibition of I_Ca_ (IC50 4.6 µM), although I_Kr_ was also sensitive [10]. In Atlantic cod (*Gadus morhua*), 1 µM phenanthrene did not change AP duration despite reducing I_Kr_, and the depolarizing currents were insensitive—partly because the concentration used was lower than in other studies [11].

For navaga (*Eleginus nawaga*), the model of the present study, fluorene suppresses I_Na_ and I_Ca_ but most strongly I_Kr_, prolonging the AP at 3 µM [12], and 3-methylphenanthrene disrupts both electrical and contractile activity [7,13]. Collectively, these findings show that the cardiotoxic effects of tricyclic PAHs are strongly species-specific, depending on the relative sensitivity and contribution of the depolarizing and repolarizing currents in each species.

However, all studies to date examining the effects of WSF or individual PAHs on the electrophysiology of fish cardiomyocytes have been conducted under acute exposure conditions. In natural environments, fish are subjected to prolonged exposure to these compounds and their mixtures. PAHs from WSF initially penetrate the organism through mucosal surfaces, and their tissue concentrations rise rapidly—within several hours—reaching levels of tens to hundreds of micrograms per kilogram of body weight. Over the following days, tissue PAH concentrations decline and stabilize at a lower steady-state level [14]. The rapid penetration of PAHs through the gills and skin into animal tissues, together with the relatively high concentrations attained during the initial days of exposure, underscores the importance of investigating the subchronic effects of WSF and its constituents. **Accordingly, the aim of the present study was to examine how prolonged (5–8 h) exposure to WSF and its most abundant tricyclic PAH constituents—phenanthrene and 3-methylphenanthrene—alters the electrical activity of ventricular cardiomyocytes in an ecologically and commercially important Arctic species, the navaga cod (*Eleginus nawaga*).**

## 2. Materials and Methods

### 2.1. Animals

All experiments were conducted at the White Sea Biological Station of Lomonosov Moscow State University (Russia). The adult navaga cod of either sex (*Eleginus nawaga*, Walbaum, 1792), an Arctic gadid species, was collected from Kandalaksha Bay of the White Sea in the vicinity of the White Sea Biological Station (Karelia, Russia; 66°19′50″ N, 33°40′06″ E). Specimens were captured using hook and line during mid-summer (July 2025), when water temperatures were 10–14 °C.

Housing, husbandry and welfare monitoring complied with the conditions approved by the MSU Bioethics Commission (animal-holding application No. 13.2, approved 16 November 2023). Fish (body mass = 96.4 ± 7.42 g, *N* = 21) were held at the Small Aquarium Facility of the White Sea Biological Station in a single 300 L tank (up to 15 individuals) supplied with continuously flowing, filtered seawater drawn from Kandalaksha Bay (exchange rate ~3 L min^−1^), so that water temperature (10 °C), salinity (26‰) and pH (8) matched ambient bay conditions; a natural photoperiod was maintained, supplemented by artificial lighting. Fish were fed lugworms (*Arenicola marina*) once daily. Upon capture, all animals were inspected for fungal infection and physical damage, and any affected individuals were returned to the wild; the remaining fish were quarantined in the holding tank for at least 7 days before experiments, during which they were monitored for signs of disease or abnormal behavior up to four times daily. No environmental enrichment was provided, and animals were not individually marked. To reduce handling stress, fish were observed visually without disturbance, and individuals showing abnormal swimming, disease or injury were excluded and returned to their natural habitat. No anesthetics or analgesics were used, as these agents would alter the ionic currents under study. Fish were euthanized by a sharp blow to the head followed by destruction of the brain (within 5–10 s of removal from the tank), in accordance with Annex IV of EU Directive 2010/63/EU, with death confirmed by cessation of opercular movement. The maximum anticipated severity was classified as moderate. Humane endpoints (marked disorientation such as listing or belly-up swimming, repeated refusal of food, visible bleeding, or extensive skin damage) were defined a priori; no adverse events or unexpected mortality occurred during holding.

No pre-registered protocol exists for this study.

### 2.2. Isolation of Cardiac Myocytes

Isolated ventricular cardiomyocytes were prepared by enzymatic dissociation, following our earlier approach for this species with minor modifications [15]. After euthanasia (see Section 2.1), the heart was quickly removed, and a cannula was inserted into the bulbus arteriosus for retrograde perfusion. The initial, nominally Ca^2+^-free perfusion medium contained the following (in mmol L^−1^): NaCl 100, KCl 10, KH_2_PO_4_·2H_2_O 1.2, MgSO_4_·7H_2_O 4, taurine 50, glucose 10, and HEPES 10, adjusted to pH 6.9. Enzymatic digestion was then initiated by switching to the same medium supplemented with collagenase type IA (0.35 mg mL^−1^), trypsin type IX (0.15 mg mL^−1^), and fatty acid-free bovine serum albumin (0.35 mg mL^−1^). These concentrations were adapted from a protocol previously optimized for navaga [12]. Collagenase type IA and trypsin were obtained from Sigma-Aldrich (St. Louis, MO, USA).

After 7–15 min of perfusion, the ventricle was separated, cut into small pieces and gently triturated to liberate single myocytes, which were kept in the isolation medium at 4 °C for up to 8 h. The effects of prolonged exposure to the water-soluble fraction (WSF) of crude oil, phenanthrene (P), and 3-methylphenanthrene (3-MP) on action potential (AP) parameters and ionic currents (I_Na_, I_Ca_, I_Kr_, I_K1_) were examined.

### 2.3. Prolonged Incubation of Cardiomyocytes with Test Substances

Myocytes isolated from each fish were divided into three to four aliquots, which were assigned to the control group and to the experimental groups (WSF, Phe, and/or 3-MP), so that cardiomyocytes from every individual contributed to several groups. This within-animal design was adopted to minimize inter-individual variability as a confounding factor, since control and treatment groups were drawn from the same isolation and recorded on the same day in an interleaved order. Allocation of aliquots to groups was randomized. Isolated ventricular myocytes were subjected to prolonged incubation with one of the following test substances: 10% water-soluble fraction (WSF) of Urals-grade crude oil, 3 μM phenanthrene (Phe), or 3 μM 3-methylphenanthrene (3-MP). Stock solutions of phenanthrene and 3-methylphenanthrene (10^−2^ M) were prepared in dimethyl sulfoxide (DMSO) and renewed every two days.

The WSF was prepared fresh on a daily basis by adding 50 μL of crude oil to 50 mL of isolation solution. The mixture was vigorously agitated for 5 min until a homogeneous suspension was formed. The resulting emulsion was then transferred to a separating funnel and allowed to settle. The lower aqueous phase, free of visible oil droplets, was collected and used for incubation. The WSF of crude oil consists predominantly of low-molecular-weight aromatic hydrocarbons, mainly di- and tricyclic PAHs (naphthalenes, phenanthrenes and their alkylated derivatives), of which phenanthrene and 3-methylphenanthrene are among the most abundant tricyclic constituents; these were therefore selected as representative PAHs. The composition of the WSF was not analyzed in the present study; however, as the same crude oil grade (Urals) and preparation procedure were used, its tricyclic PAH content can be taken from our previous work [16], in which the 10% WSF was characterized by gas chromatography–mass spectrometry. In that study, sixteen tricyclic compounds were detected (two of them unsubstituted), with a total concentration of ~3.2 µg L^−1^; the unsubstituted tricyclic PAHs corresponded to ~4 nM but represented only 22.3% of the total tricyclic mass. Taking into account the unquantified di- and tetracyclic PAHs, the total concentration of dissolved PAHs capable of acting on ion channels in the 10% WSF is estimated in the tens of nM, not exceeding ~100 nM.

Cardiomyocytes were incubated with the test substances for 5–8 h (within the storage limit of 8 h, see Section 2.2) prior to electrophysiological recordings.

Cell viability was assessed morphologically and functionally rather than by separate biochemical assays. Prior to each recording, cardiomyocytes were visually inspected under the microscope, and only cells displaying the normal morphology of fish ventricular myocytes—an elongated rod shape, smooth and even surface membranes without blebbing, and clear cross-striations—were selected for experiments. Cells showing rounding, membrane blebbing, granularity, or loss of striation were rejected. In addition, in current-clamp experiments, only cells with a stable resting membrane potential more negative than −70 mV were accepted, and in voltage-clamp experiments, only cells with low leak current (<100 pA) and series resistance (<10 MΩ) were used, providing a functional confirmation of membrane integrity in the recorded subset. Because control myocytes were incubated in parallel for the same 5–8 h period under the within-animal design, any time-dependent deterioration was applied equally across all groups and cannot account for the treatment-specific differences observed.

The concentrations used here (3 µM for individual PAHs and 10% WSF) were selected to match those employed in previous acute electrophysiological studies on navaga and other fish species [7,12,16], allowing direct comparison between acute and subchronic effects. Although a precise environmental equivalent is difficult to define—tissue PAH burdens depend on exposure duration, species and lipid content—3 µM phenanthrene is within the same order of magnitude as the tens-to-hundreds of µg kg^−1^ tissue concentrations reported in fish chronically exposed to dispersed oil [14], and 10% WSF represents a moderate dilution of an acutely toxic mixture. These doses should therefore be regarded as environmentally relevant in magnitude rather than as exact field concentrations.

### 2.4. Voltage-Clamp and Current-Clamp Recordings

For recordings, an aliquot of the myocyte suspension was placed in a 150 μL chamber and perfused continuously (~1.5 mL min^−1^) with a standard K^+^-based physiological saline containing the following (in mmol L^−1^): NaCl 150, KCl 3.5, NaH_2_PO_4_ 0.4, MgSO_4_ 1.5, CaCl_2_ 1.8, glucose 10, and HEPES 10, adjusted to pH 7.3 with NaOH at room temperature. Bath temperature was held at 12 °C with a CL-100 controller (Warner Instruments LLC., Holliston, MA, USA).

Whole-cell patch-clamp recordings were made with an Axopatch 200A amplifier (Molecular Devices, San Jose, CA, USA) driven by WinWCP v.5.7.7 (University of Strathclyde, Glasgow, UK). Recording pipettes (2.0–3.0 MΩ) were pulled from borosilicate capillaries (Sutter Instruments, Novato, CA, USA), and pipette capacitance, series resistance and whole-cell capacitance were compensated in each experiment. Pipette capacitance, series resistance, and whole-cell capacitance were routinely compensated.

For AP and K^+^ current recordings, the external K^+^-based physiological solution described above was used in conjunction with pipettes filled with a K^+^-based intracellular solution containing the following (in mmol L^−1^): KCl 140, MgCl_2_ 1, EGTA 5, MgATP 4, Na_2_GTP 0.3, and HEPES 10, adjusted to pH 7.2 with KOH. During current-clamp experiments, only cells exhibiting a stable resting membrane potential (RMP) more negative than −70 mV were selected for analysis. Myocytes were stimulated with 1 ms depolarizing pulses delivered at 0.2 Hz. Stimulus amplitude ranged from 0.2 to 2 nA, depending on the excitability of individual cells. Recordings were initiated only after a stable RMP and AP configuration had been established (approximately 5 min following membrane rupture).

The rapid delayed rectifier K^+^ current (I_Kr_, K_v_11.1, ERG) was evoked with a two-pulse protocol from a −80 mV holding level: a 2 s conditioning step to potentials between −80 and +60 mV (20 mV increments) was followed by a 2 s step to −20 mV, and I_Kr_ was measured as the tail current at −20 mV. The E-4031-sensitive component was taken as the specific current, obtained by subtracting traces recorded in 2 μM E-4031 (Tocris Bioscience, Bristol, UK) from control traces. The inward rectifier current (I_K1_, K_ir_2.x) was elicited from −80 mV by a 1 s ramp from +60 to −120 mV applied every 10 s, and was defined as the Ba^2+^-sensitive component (subtraction of traces in 2 mM BaCl_2_; Sigma-Aldrich, St. Louis, MO, USA). To isolate the K^+^ currents, nifedipine (20 μM; Sigma-Aldrich, St. Louis, MO, USA) was added to the bath to block I_Ca_ [15]. During I_Kr_ recordings, I_Na_ was inactivated by the conditioning pulse, and during I_K1_ ramps by the depolarized starting potential, so that Na^+^ current did not contaminate the measurements.

For I_Na_ (Na_v_1.5), extracellular Na^+^ was lowered below physiological levels to reduce the driving force on Na^+^ and preserve voltage control. The bath solution contained the following (in mmol L^−1^): NaCl 20, CsCl 120, MgCl_2_ 1, CaCl_2_ 0.5, glucose 10, and HEPES 10, adjusted to pH 7.7 with CsOH. The pipette solution for I_Na recordings comprised the following (in mmol L^−1^): NaCl 5, CsCl 130, MgCl_2_ 1, EGTA 5, Mg_2_ATP 5, and HEPES 5, adjusted to pH 7.2 with CsOH [13]. Nifedipine (20 μM) was present in the solution to block I_Ca,L_, and ~60% of the series resistance was compensated to capture the fast, large Na^+^ current faithfully. Currents were elicited with a two-step protocol: 300 ms steps from −120 mV to between −120 and +60 mV (10 mV increments) yielded the current–voltage (I–V) relationship and the steady-state (SS) activation, the latter obtained by fitting normalized conductance (G/G_max_) versus voltage to a Boltzmann function:

(1) y=1(1+eV−V50S)

where V is the membrane potential, V_50_ is the half-activation potential, and S is the slope factor. Conductance was calculated as G_Na_ = I_Na_/(V − V_rev_), where I_Na_ is the peak current at a given membrane potential V and V_rev_ is the reversal potential. G_Kr_ = I_Kr_/I_Kr,max_, where I_Kr_ is the tail current at a given membrane potential and I_Kr,max_ is the maximum tail current. SS inactivation was determined from the normalized current (I/I_max_) elicited by a second 200 ms test pulse to −20 mV, plotted as a function of the preceding conditioning potential, and fitted to a Boltzmann function with a negative slope (−S).

For recording of the L-type Ca^2+^ current (I_Ca,L_, Ca_v_1.2), K^+^ in the external saline was replaced by equimolar Cs^+^, and the pipette contained (in mmol L^−1^): CsCl 130, MgCl_2_ 1, EGTA 5, tetraethylammonium chloride 15, MgATP 4, Na_2_GTP 0.3, and HEPES 10, adjusted to pH 7.2 with CsOH. The I–V relationship was obtained with 250 ms pulses from a −40 mV holding potential to between −40 and +50 mV (10 mV increments); holding at −40 mV fully inactivated I_Na_, isolating the Ca^2+^ current.

Data were analyzed using Clampfit 11.3 software (Molecular Devices, San Jose, CA, USA). All current amplitudes were normalized to cell capacitance and expressed as current density (pA pF^−1^). The activation time constant of the I_Kr_ tail current, the inactivation time constant of I_Na_ and the inactivation time constant of I_Ca,L_ were obtained by fitting the decaying phase of the current trace to a monoexponential function using the Chebyshev transformation tool in Clampfit 11.3.

### 2.5. Statistics

Data are presented as means ± SEM. Blinding was not performed during recording. All statistical analyses were conducted using GraphPad Prism v.8 software (GraphPad Software Inc., Boston, MA, USA). The normality of data distribution was assessed using the Shapiro–Wilk test prior to all comparisons. When the assumption of normality was met, parametric tests were applied; otherwise, the corresponding non-parametric tests were used. For one-way comparisons, homogeneity of variances was assessed by the Brown–Forsythe test. For the nested and mixed-effects analyses, the assumption of homo-(hetero-)scedasticity was verified by inspection of residual plots. Comparisons of AP parameters, as well as I_Na_ and I_Ca,L_ inactivation time constants or the I_Kr_ activation time constant, were performed using nested one-way ANOVA with Dunnett post hoc test. Current–voltage relationships for I_Kr_, I_Ca,L_ and I_Na_ between groups were compared using mixed-effects model ANOVA (MM-ANOVA) with Geisser–Greenhouse correction, followed by post hoc tests indicated in the figure legends. Steady-state activation and inactivation parameters of I_Na_ between groups were compared using one-way ANOVA. Statistical significance was defined as *p* < 0.05. Sample sizes, including the number of individual myocytes (*n*) and the number of fish (*N*, experimental unit), are reported in the corresponding figure legends. Sample sizes were consistent with previous patch-clamp studies on this species [7,12,13,15,16,17]. A sensitivity analysis (G*Power 3.1; two-tailed α = 0.05, power = 0.80) was conducted at the level of individual fish (the experimental unit), using the between-fish standard deviation estimated from the data. For the comparison between control (*N* = 13) and the smallest treatment group showing a significant effect on AP duration (Phe, *N* = 9), the design was sufficient to detect a standardized effect size of Cohen’s d ≈ 1.27, corresponding to a difference of approximately 17% of the control APD50. The observed effects on APD50 (d ≈ 1.7 for Phe and 2.1 for 3-MP) exceeded this threshold.

## 3. Results

The prolonged effects of 10% water-soluble fraction of crude oil (WSF), 3 µM 3-methylphenanthrene (3-MP), and 3 µM phenanthrene (Phe) were investigated by prolonged incubation (5–8 h) of cardiomyocytes with one of these compounds, followed by electrophysiological recordings in the absence of the test substances in the recording solutions.

### 3.1. The Effect of Prolonged Incubation with WSF, P and 3MP on AP Parameters

**Figure 1:**
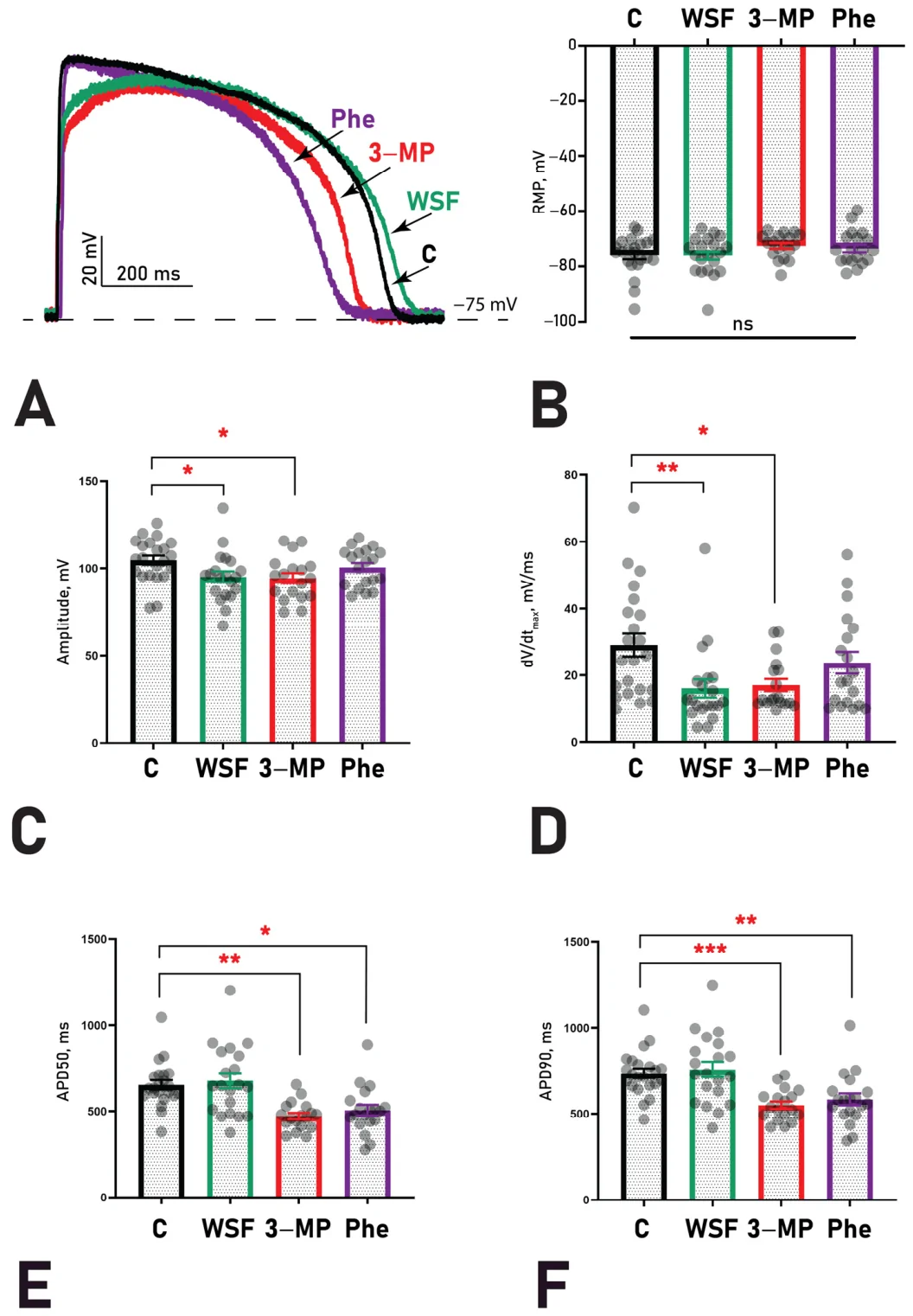
Effects of prolonged incubation of ventricular cardiomyocytes of the navaga cod (*Eleginus nawaga*) with 10% water-soluble fraction of crude oil (WSF), 3 µM phenanthrene (Phe), or 3 µM 3-methylphenanthrene (3-MP) on action potential (AP) parameters. (**A**) Representative AP recordings under control conditions and following prolonged incubation with WSF, Phe, or 3-MP. (**B**) Effect of prolonged incubation with WSF, P, or 3-MP on resting membrane potential. (**C**) Effect of prolonged incubation with WSF, Phe, or 3-MP on AP amplitude. (**D**) Effect of prolonged incubation with WSF, Phe, or 3-MP on the maximum rate of depolarization. (**E**) Effect of prolonged incubation with WSF, Phe, or 3-MP on AP duration at 50% repolarization (APD50). (**F**) Effect of prolonged incubation with WSF, Phe, or 3-MP on AP duration at 90% repolarization (APD90). Data are presented as mean ± SEM for *n* = 22 (*N* = 13, control), *n* = 20 (*N* = 6, WSF), *n* = 18 (*N* = 10, 3-MP), *n* = 19 (*N* = 9, P). *, **, ***—*p* < 0.05; *p* < 0.01, *p* < 0.001 nested one-way ANOVA with Dunnett’s post hoc test.

WSF, P, and 3-MP exerted significant effects on action potential (AP) parameters of ventricular cardiomyocytes of the navaga cod (*Eleginus nawaga*) upon prolonged exposure (Figure 1A). Incubation with WSF and 3-MP, but not Phe, resulted in a statistically significant reduction in AP amplitude from 104.8 ± 2.65 mV under control conditions to 95.1 ± 3.23 mV (95% CI −19.2 to −0.08; *p* < 0.05) and 94.4 ± 2.95 mV (95% CI −20.2 to −0.56; *p* < 0.05), respectively (Figure 1C). A comparable effect was observed for the maximum rate of depolarization: incubation with WSF and 3-MP led to a statistically significant decrease in this parameter from 29.0 ± 3.47 mV/ms under control conditions to 16.1 ± 2.68 mV/ms (95% CI −22.9 to −2.97; *p* < 0.01) and 17.1 ± 1.81 mV/ms (95% CI −22.2 to −1.70; *p* < 0.05), respectively (Figure 1D).

AP duration at 50% repolarization (APD50) and 90% repolarization (APD90) were reduced following prolonged exposure to Phe and 3-MP, but not WSF (Figure 1E,F). Specifically, APD50 decreased from 651.1 ± 27.7 ms under control conditions to 506.3 ± 31.2 ms (95% CI −259 to −30.7; *p* < 0.05) and 472.1 ± 19.0 ms (95% CI −294 to −62.5; *p* < 0.01) after incubation with Phe or 3-MP, respectively. APD90 decreased from 734 ± 28.3 ms under control conditions to 583.3 ± 34.4 ms (95% CI −263 to −38.2; *p* < 0.01) and 550 ± 34.4 ms (95% CI −298 to −69.8; *p* < 0.001) after incubation with Phe or 3-MP, respectively.

None of the compounds tested affected the resting membrane potential following prolonged incubation (Figure 1B).

### 3.2. The Effect of Prolonged Incubation with WSF, P and 3MP on Ion Currents

**Figure 2:**
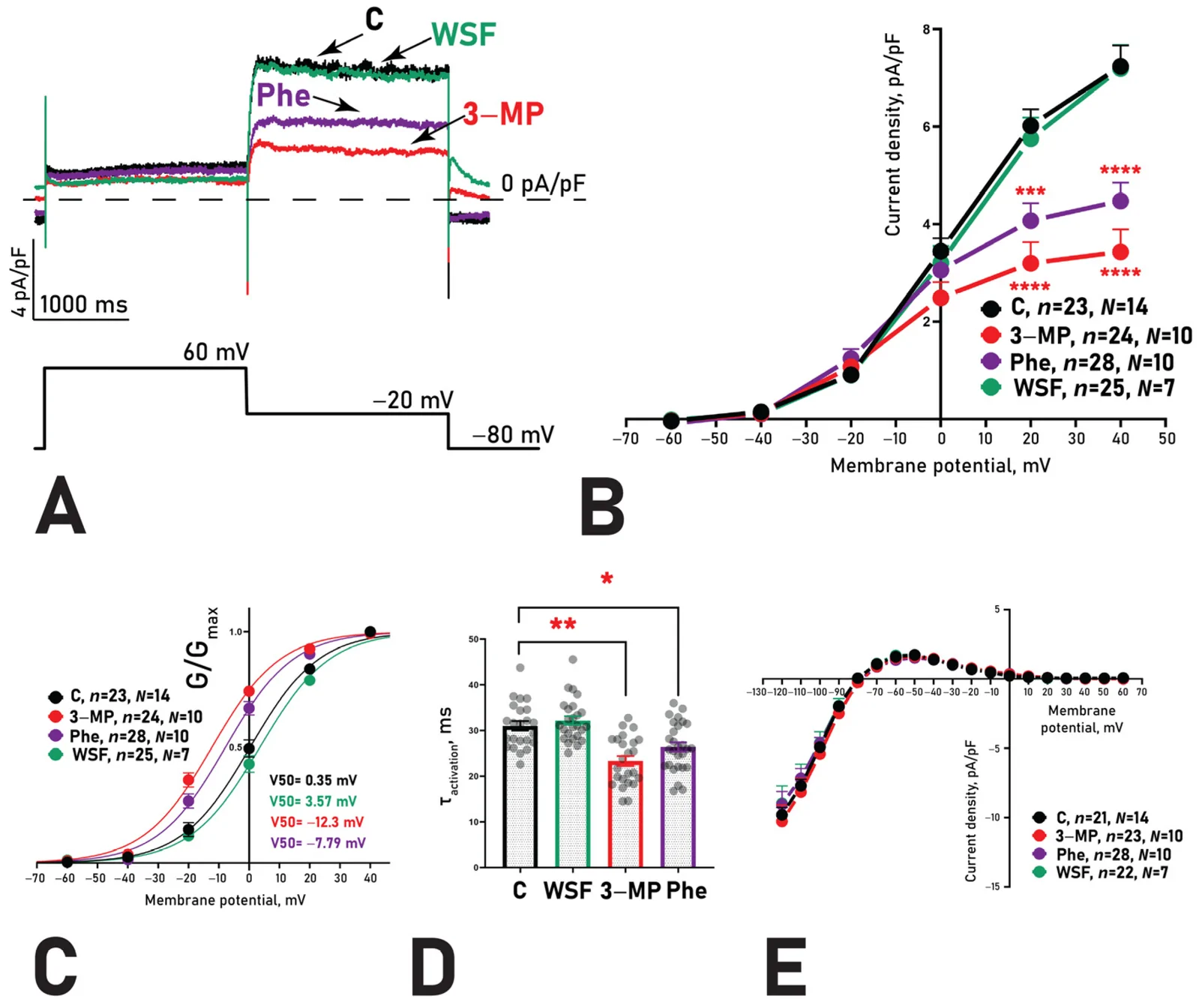
Effects of prolonged incubation of ventricular cardiomyocytes of the navaga cod (*Eleginus nawaga*) with 10% water-soluble fraction of crude oil (WSF), 3 µM phenanthrene (Phe), or 3 µM 3-methylphenanthrene (3-MP) on the potassium currents, I_Kr_ and I_K1_. (**A**) Representative I_Kr_ recordings under control conditions and following prolonged incubation with WSF, Phe, or 3-MP. The inset below indicates the corresponding voltage-clamp protocol. (**B**) Effect of prolonged incubation with WSF, Phe, or 3-MP on the current–voltage relationship of the I_Kr_ tail current. ***, ****—*p* < 0.001; *p* < 0.0001, mixed-effects model ANOVA (MM-ANOVA) with Dunnett’s post hoc test compared with control values. (**C**) Effect of prolonged incubation with WSF, 3-MP, or Phe on the steady-state activation parameters of I_Kr_. (**D**) Effect of prolonged incubation with WSF, 3-MP, or Phe on the activation time constant of I_Kr_. *, **—*p* < 0.05, 0.01 nested one-way ANOVA. (**E**) Effect of prolonged incubation with WSF, 3-MP, or Phe on the current–voltage relationship of the inward rectifier potassium current, I_K1_. Data are presented as mean ± SEM. The numbers of cardiomyocytes (*n*) and fish (*N*) are indicated in the figure.

Prolonged incubation of ventricular cardiomyocytes with Phe and 3-MP, but not with WSF, resulted in a statistically significant reduction in the density of the rapid delayed rectifier potassium current, I_Kr_ (Figure 2). All tested substances changed the V50 parameter of steady-state activation of I_Kr_. Phe and 3-MP shifted V50 to the more negative potentials, and WSF shifted it to the more positive potential (see Table 1). In addition to the reduction in current density, prolonged incubation with Phe and 3-MP accelerated the activation of the I_Kr_ tail current: τ_act_ at +40 mV decreased from 31.0 ± 1.05 ms under control conditions to 26.4 ± 1.00 ms (Phe; *p* < 0.05) and 23.3 ± 1.10 ms (3-MP; *p* < 0.01), whereas WSF had no effect (32.2 ± 0.94 ms).

The inward rectifier potassium current, I_K1_, was resistant to prolonged exposure to all compounds tested (Figure 2E).

**Figure 3:**
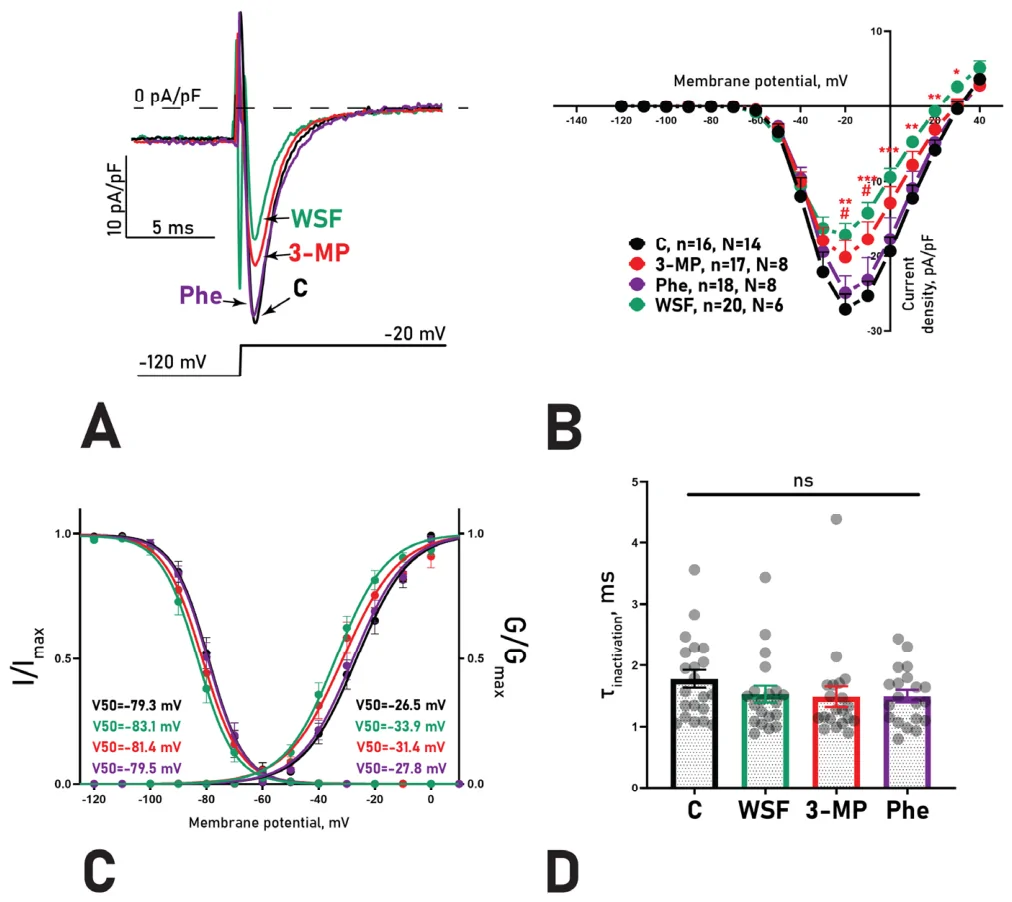
Effects of prolonged incubation of ventricular cardiomyocytes of the navaga cod (*Eleginus nawaga*) with 10% water-soluble fraction of crude oil (WSF), 3 µM phenanthrene (Phe), or 3 µM 3-methylphenanthrene (3-MP) on the fast sodium current, I_Na_. (**A**) Representative I_Na_ recordings at a depolarizing step to −20 mV under control conditions and following prolonged incubation with WSF, Phe, or 3-MP. The inset below indicates the voltage-clamp protocol. (**B**) Effect of prolonged incubation with WSF, 3-MP, or Phe on the current–voltage relationship of I_Na_. (**C**) Effect of prolonged incubation with WSF, 3-MP, or Phe on the steady-state activation and inactivation parameters of I_Na_. (**D**) Effect of prolonged incubation with WSF, 3-MP, or Phe on the inactivation time constant of I_Na_. Data are presented as mean ± SEM. The number of cardiomyocytes (*n*) and fish (*N*) are indicated in the figure. *, **, ***—*p* < 0.05; *p* < 0.01; *p* < 0.001 for comparison of I_Na_ following prolonged incubation with WSF versus control values. #—*p* < 0.05 for comparison of I_Na_ following prolonged incubation with 3-MP versus control values. All comparisons were performed using mixed-effects model ANOVA (MM-ANOVA) with Dunnett’s post hoc test.

Prolonged incubation of ventricular cardiomyocytes with WSF and 3-MP, but not with Phe, resulted in a statistically significant decrease in the density of the fast sodium current, I_Na_ (Figure 3A,B), as well as a leftward shift of both the steady-state activation and inactivation curves (Figure 3C, Table 2). These findings account for the observed reductions in AP amplitude and maximum rate of depolarization following prolonged exposure to WSF and 3-MP, but not Phe.

The inactivation kinetics of I_Na_ were unchanged: τ_inact_ at −20 mV did not differ significantly between control (1.78 ± 0.15 ms) and any treatment group (WSF, 1.53 ± 0.14 ms; 3-MP, 1.49 ± 0.17 ms; Phe, 1.50 ± 0.10 ms, see Figure 3D).

**Figure 4:**
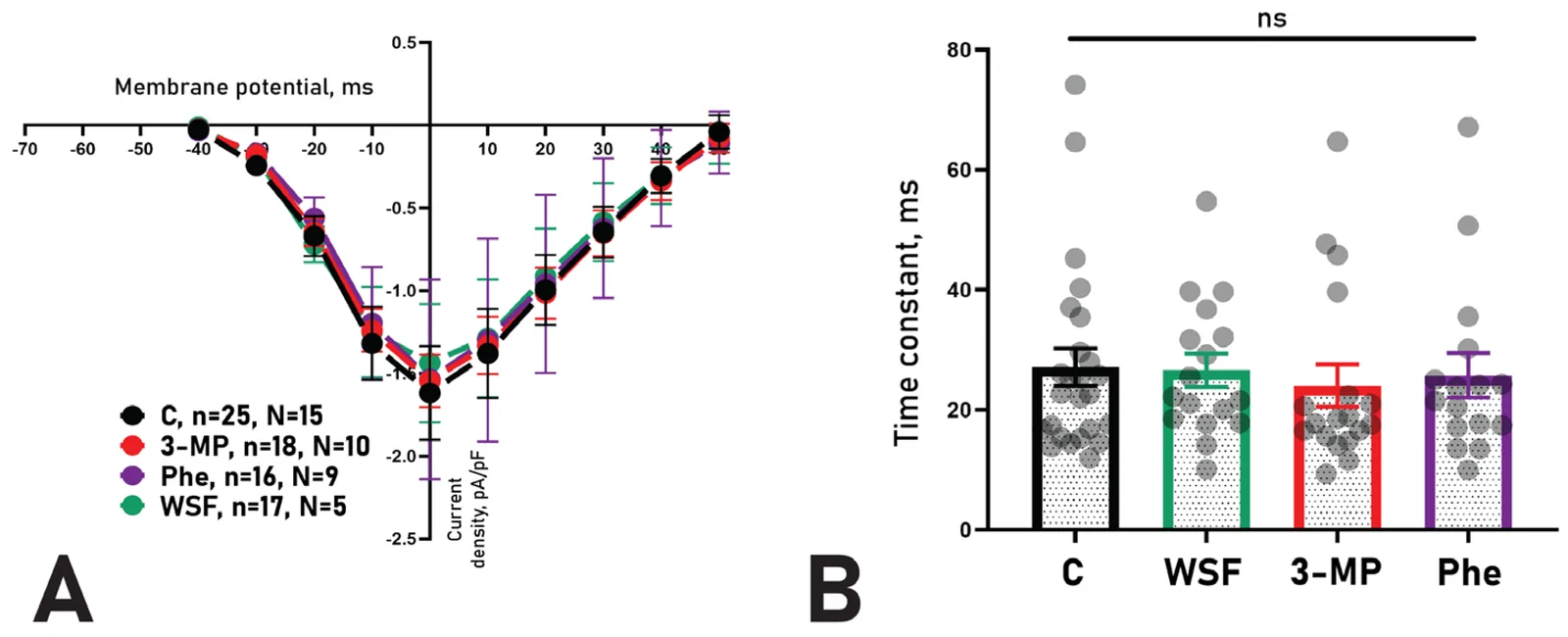
Effects of prolonged incubation of ventricular cardiomyocytes of the navaga cod (*Eleginus nawaga*) with 10% water-soluble fraction of crude oil (WSF), 3 µM phenanthrene (Phe), or 3 µM 3-methylphenanthrene (3-MP) on the L-type calcium current, I_Ca,L_. (**A**) Effect of prolonged incubation with WSF, 3-MP, or Phe on the current–voltage relationship of I_Ca,L_. (**B**) Effect of prolonged incubation with WSF, 3-MP, or Phe on the inactivation time constant of I_Ca,L_. Mixed-effects model ANOVA (MM-ANOVA) with Dunnett’s post hoc test was used for current density comparison and nested one-way ANOVA was used for time constant comparison. The numbers of cardiomyocytes (*n*) and fish (*N*) are indicated in the figure.

Finally, the parameters of the L-type calcium current were not altered following prolonged incubation with any of the compounds tested (Figure 4).

## 4. Discussion

In this study, we investigated the effects of prolonged incubation of ventricular cardiomyocytes from the navaga cod (*Eleginus nawaga*) with the water-soluble fraction of crude oil (WSF) and individual polycyclic aromatic hydrocarbons (PAHs) on the electrical activity parameters and ionic currents of these cells.

The water-soluble fraction of crude oil is a solution composed predominantly of di- and tricyclic polycyclic aromatic hydrocarbons, among which phenanthrene (Phe) and its methylated derivative, 3-methylphenanthrene (3-MP), are the most abundant constituents. These same compounds tend to bioaccumulate in the tissues of fish inhabiting polluted waters. For this reason, Phe and 3-MP were selected as representative PAHs for the present experiments [18,19,20,21,22,23,24].

### 4.1. Prolonged Incubation with WSF or PAHs Are Detrimental to Fish Ventricular Cardiomyocytes Electrophysiology

Incubation of ventricular cardiomyocytes with the compounds under investigation resulted in substantial alterations in ionic currents of these cells and, consequently, in the shape of action potential (AP). Under the influence of the WSF, both the AP amplitude and the maximum rate of depolarization were reduced. Phe promoted a decrease in AP duration, whereas 3-MP reduced the AP amplitude, the maximum rate of depolarization, and the AP duration simultaneously.

The reduction in the amplitude of the fast sodium current (I_Na_) in cardiomyocytes exposed to WSF and 3-MP, which leads to decreased AP amplitude and maximum rate of depolarization, is proarrhythmogenic. I_Na_ is a key determinant of the conduction velocity (θ) across the myocardium. Slowing of θ constitutes an important substrate for the development of reentrant excitation, as disruption of conduction synchrony increases the probability of pathological circulation of the excitation wave and functional conduction block within cardiac tissue [25]. In clinical and experimental models, a reduction in I_Na_ resulting from sodium channels dysfunction is associated with conduction slowing, decreased excitability, and an elevated risk of both ventricular and atrial arrhythmias [26]. Thus, the PAH-induced decrease in I_Na_ amplitude may contribute to the establishment of an electrophysiological substrate favorable for arrhythmogenesis, particularly when combined with other insults to the conduction system.

A decrease in AP duration under Phe and 3-MP exposure leads to a reduction in the amount of Ca^2+^ ions entering cardiomyocytes during the AP plateau phase. Consequently, the quantity of calcium ions serving as “trigger” calcium for the initiation of excitation–contraction coupling in these cells is diminished. This, in turn, reduces myocardial contractility, thereby decreasing the stroke work produced by the ventricle during a single cardiac cycle [27,28].

### 4.2. The Effects of Prolonged Incubation Differ from Those of Acute Exposure

The effects of prolonged incubation of ventricular cardiomyocytes with the water-soluble fraction of crude oil or individual tricyclic PAHs differed from those observed under acute exposure to the same compounds. Acute exposure to 10% WSF caused AP prolongation through suppression of I_Kr_ and partial inhibition of I_Ca_, while I_Na_ remained unchanged [16]. The estimated PAH concentration in the 10% WSF (tens of nM) is several orders of magnitude lower than that required for the direct effects of individual tricyclic PAHs on I_Na_, yet prolonged incubation with the WSF clearly altered I_Na_, reducing AP amplitude and the maximum rate of depolarization. Importantly, this reduction was not accompanied by any change in the inactivation kinetics of I_Na_, indicating a decrease in current amplitude rather than accelerated channel inactivation.

Phenanthrene at a concentration of 3 μM acutely suppressed only I_Kr_ without altering AP parameters [12], whereas prolonged incubation shortened AP duration. This shortening cannot be explained by the reduction in I_Kr_ amplitude alone; rather, prolonged incubation with Phe accelerated the activation of the I_Kr_ tail current and shifted its steady-state activation toward more negative potentials, both of which allow the repolarizing current to develop earlier during the AP and thereby shorten its duration despite the decreased current density.

In the case of 3-methylphenanthrene, acute exposure was accompanied by pronounced changes in AP morphology due to marked suppression of I_Kr_, partial inhibition of I_Na_, and acceleration of I_Ca,L_ inactivation [7]. By contrast, prolonged incubation with 3-methylphenanthrene combined a reduction in I_Kr_ and I_Na_ amplitudes with the same kinetic changes in I_Kr_—an acceleration of tail-current activation together with a hyperpolarizing shift in its steady-state activation—which together account for the decrease in AP amplitude and the shortening of AP duration.

What accounts for the differences between the effects of acute exposure and those of prolonged incubation with the compounds? During acute exposure (up to 10 min), the observed effects are thought to be mediated primarily by direct interaction of the compounds with channel proteins and possible activation of the phosphatidylinositol or adenylate cyclase signaling cascades. During prolonged incubation of isolated cells, the emphasis shifts toward alterations in intracellular signaling cascades associated with the regulation of gene transcription and the response to oxidative stress and inflammation, such as the MAPK cascade and pathways mediated through NF-κB [29,30].

Specifically, exposure of various cell cultures to Phe for 12–24 h has been shown to increase the content of reactive oxygen species (ROS) and activate the cellular antioxidant defense system [31,32,33]. ROS are capable of altering the electrical activity of cardiomyocytes by reducing the amplitude of major ionic currents; such effects have been demonstrated for I_Kr_ and I_Na_ in mammalian cardiomyocytes [34,35,36,37]. Notably, I_Kr_ and I_Na_ are precisely the two currents suppressed by prolonged incubation in the present study, whereas I_Ca,L_ and I_K1_—currents for which a comparable ROS-mediated reduction is less consistently reported—remained unaffected. This correspondence suggests that ROS accumulation during prolonged PAH exposure is a plausible mediator of the reductions in I_Kr_ and I_Na_ that we observed. ROS can further activate the MAPK cascade and NF-κB. Although the effects of these cascades on ionic current density have not been examined directly in fish ventricular cardiomyocytes, in neonatal rat cardiomyocytes their activation decreased I_to_ density and prolonged AP duration [38]; a contribution of such signaling to the AP-duration changes seen here, therefore, cannot be excluded. Activation of NF-κB has also been shown to increase L-type calcium current density in rat ventricular cardiomyocytes [39]; the absence of any change in I_Ca,L_ in our experiments indicates that, if this pathway is engaged in navaga cardiomyocytes, its effect on I_Ca,L_ is either minor or offset by other processes. Taken together, the pattern of currents affected in the present study—a reduction in I_Kr_ and I_Na_ without changes in I_Ca,L_ or I_K1_—is broadly consistent with ROS- and MAPK/NF-κB-mediated modulation, although these mechanisms were not measured directly and remain to be tested experimentally.

Notably, prolonged PAH exposure in several studies disrupted calcium homeostasis in cardiomyocytes in a manner distinct from that observed during acute exposures. Whereas in acute experiments phenanthrene, retene, and fluoranthene enhanced SERCA activity in fish, prolonged exposure resulted in suppression of the expression of the *atp2a2a* gene encoding the SERCA2A protein [40]. The cytoplasmic calcium accumulation accompanying SERCA inhibition may promote early afterdepolarizations and ectopic activity through activation of the NCX exchanger, as well as induce apoptosis [41,42,43].

Finally, tricyclic PAHs are capable of stimulating the production of proinflammatory interleukins, particularly IL-6, both in cell cultures and upon whole-organism exposure [33,44,45,46]. Electrophysiological studies have demonstrated that IL-6 suppresses the rapid delayed rectifier potassium current (I_Kr_) in cardiomyocytes and in HEK-293T cells expressing hERG [47,48], as well as the slow delayed rectifier potassium current (I_Ks_) [48]. Data regarding the effect of IL-6 on calcium current amplitude are inconsistent: in guinea pig studies, IL-6 decreased I_Ca,L_ amplitude [49], whereas in mouse cardiomyocytes, IL-6 increased the amplitude of this current [50]. Thus, PAH-stimulated release of proinflammatory cytokines may have contributed to the observed alterations in the electrical activity characteristics and parameters of the major ionic currents in cardiomyocytes.

In summary, we hypothesize that WSF- or individual PAH-induced production of ROS and activation of the MAPK and NF-κB signaling cascades, as well as elevation of cytoplasmic calcium levels, are capable of significantly altering ionic current densities in navaga ventricular cardiomyocytes; however, the specific effects are dependent on the compound under investigation.

## 5. Conclusions

The present study demonstrates for the first time that prolonged exposure of isolated ventricular cardiomyocytes of the navaga cod (*Eleginus nawaga*) to the water-soluble fraction of crude oil and its principal tricyclic PAH constituents—phenanthrene and 3-methylphenanthrene—produces qualitatively distinct electrophysiological alterations compared with acute exposure to the same compounds. Prolonged incubation differentially affected the fast sodium current and the rapid delayed rectifier potassium current, leading to compound-specific changes in action potential morphology that are potentially proarrhythmogenic and detrimental to cardiac contractility. These findings underscore the importance of incorporating subchronic exposure paradigms into the assessment of PAH cardiotoxicity in fish, as acute experiments alone may underestimate or mischaracterize the true electrophysiological impact of these ubiquitous pollutants. Future studies should elucidate the intracellular signaling mechanisms—including reactive oxygen species production and MAPK/NF-κB pathway activation—that mediate the transition from acute to subchronic cardiotoxic responses in fish cardiomyocytes.

This study has several limitations that should be considered when interpreting the results. Most importantly, our experiments were performed on isolated ventricular cardiomyocytes rather than on the intact heart or whole organism. While this reductionist approach allows the direct measurement of ionic currents and their contribution to the action potential, it removes the cell from its native context, including electrical coupling between myocytes, neurohumoral regulation, and the systemic and compensatory responses that shape cardiac function. Consequently, the response of the whole animal to chronic PAH exposure is likely to differ substantially from that observed at the single-cell level, and the present findings cannot be directly extrapolated to cardiac performance in the intact fish.

## Figures and Tables

**Table 1:** Effects of prolonged incubation of ventricular cardiomyocytes of the navaga cod (*Eleginus nawaga*) with 10% water-soluble fraction of crude oil (WSF), 3 µM phenanthrene (Phe), or 3 µM 3-methylphenanthrene (3-MP) on steady-state activation parameters of the I_Kr_. **, ****—*p* < 0.01; *p* < 0.0001 compared with control values. Comparisons were performed using one-way ANOVA.

Group	Steady-State Activation Parameters	
	V50, mV	Slope
Control	0.35 ± 0.78	11.3 ± 0.70
10% WSF	3.57 ± 0.75 ** (95% CI −5.76–−0.73)	11.5 ± 0.67
3 µM 3-MP	−12.2 ± 0.65 **** (95% CI 10.0–15.1)	11.3 ± 0.56
3 µM Phe	−7.79 ± 0.69 **** (95% CI 5.67–10.6)	11.5 ± 0.60

**Table 2:** Effects of prolonged incubation of ventricular cardiomyocytes of the navaga cod (*Eleginus nawaga*) with 10% water-soluble fraction of crude oil (WSF), 3 µM phenanthrene (Phe), or 3 µM 3-methylphenanthrene (3-MP) on steady-state activation and inactivation parameters of the fast sodium current, I_Na_. *, ****—*p* < 0.05; *p* < 0.0001 compared with control values. Comparisons were performed using one-way ANOVA.

Group	Steady-State Activation Parameters		Steady-State Inactivation Parameters	
	V50, mV	Slope	V50, mV	Slope
Control	−26.5 ± 0.62	9.40 ± 0.56	−79.3 ± 0.56	−6.34 ± 0.49
10% WSF	−33.9 ± 0.57 **** (95% CI 5.24–9.54)	9.13 ± 0.50	−83.1 ± 0.55 **** (95% CI 1.92–5.75)	−6.80 ± 0.56
3 µM 3-MP	−31.4 ± 0.77 **** (95% CI −0.98–3.43)	10.0 ± 0.69	−81.4 ± 0.65 * (95% CI 0.19–4.17)	−6.40 ± 0.43
3 µM Phe	−27.8 ± 0.58	9.40 ± 0.51	−79.5 ± 0.55	−6.60 ± 0.47

## Data Availability

The original data presented in the study are openly available at https://shorturl.at/NMhiR (accessed on 25 July 2026) (DOI: 10.5281/zenodo.20763230).

## Abbreviations

The following abbreviations are used in this manuscript:

3-MP	3-Methylphenanthrene
AP	Action Potential
APD	Action Potential Duration
APD50	Action Potential Duration at 50% Repolarization
APD90	Action Potential Duration at 90% Repolarization
DMSO	Dimethyl Sulfoxide
EGTA	Ethylene Glycol-bis(β-aminoethyl Ether)-N,N,N′,N′-Tetraacetic Acid
GTP	Guanosine Triphosphate
HEPES	4-(2-Hydroxyethyl)-1-piperazineethanesulfonic Acid
hERG	Human Ether-à-go-go-Related Gene
I_Ca,L_	L-type Calcium Current
I_K1_	Inward Rectifier Potassium Current
I_Kr_	Rapid Delayed Rectifier Potassium Current
I_Ks_	Slow Delayed Rectifier Potassium Current
IL-6	Interleukin-6
I_Na_	Fast Sodium Current
I_to_	Transient Outward Potassium Current
I–V	Current–Voltage Relationship
MAPK	Mitogen-Activated Protein Kinase
MgATP	Magnesium Adenosine Triphosphate
Na_2_GTP	Disodium Guanosine Triphosphate
NCX	Sodium–Calcium Exchanger
NF-κB	Nuclear Factor Kappa-Light-Chain-Enhancer of Activated B Cells
PAH	Polycyclic Aromatic Hydrocarbon
Phe	Phenanthrene
RM-ANOVA	Repeated-Measures Analysis of Variance
RMP	Resting Membrane Potential
ROS	Reactive Oxygen Species
SEM	Standard Error of the Mean
SERCA	Sarco(endo)plasmic Reticulum Calcium ATPase
SERCA2A	Sarco(endo)plasmic Reticulum Calcium ATPase Isoform 2A
SS	Steady-State
WSF	Water-Soluble Fraction of Crude Oil
